# Office workers' perspectives on physical activity and sedentary behaviour: a qualitative study

**DOI:** 10.1186/s12889-022-13024-z

**Published:** 2022-03-30

**Authors:** Lorraine L. Landais, Judith G. M. Jelsma, Idske R. Dotinga, Danielle R. M. Timmermans, Evert A. L. M. Verhagen, Olga C. Damman

**Affiliations:** 1 Department of Public and Occupational Health, Amsterdam UMC location Vrije Universiteit Amsterdam, Boelelaan, 1117 Amsterdam, The Netherlands; 2 Amsterdam Collaboration On Health & Safety in Sports, Department of Public and Occupational Health, Amsterdam Movement Sciences, UMC, Vrije Universiteit Amsterdam, Amsterdam, The Netherlands

**Keywords:** Office workers, Perspective, Physical activity, Sedentary behaviour, Qualitative research, Beliefs, Values

## Abstract

**Background:**

Office workers spend a significant part of their workday sitting. Interventions that aim to reduce sedentary behaviour and increase physical activity might be more effective if greater attention is paid to individual perspectives that influence behavioural choices, including beliefs and values. This study aimed to gain insight into office workers' perspectives on physical activity and sedentary behaviour.

**Methods:**

Sixteen Dutch office workers (50% female) from different professions participated in semi-structured face-to-face interviews in March 2019. To facilitate the interviews, participants received a sensitizing booklet one week before the interview. The booklet aimed to trigger them to reflect on their physical activity and sedentary behaviour and on their values in life. All interviews were audiotaped, transcribed verbatim and coded following codebook thematic analysis.

**Results:**

Six themes were identified: 1) beliefs about health effects are specific regarding physical activity, but superficial regarding sedentary behaviour; 2) in addition to ‘health’ as a value, other values are also given priority; 3) motivations to engage in physical activity mainly stem from prioritizing the value ‘health’, reflected by a desire to both achieve positive short/mid-term outcomes and to prevent long-term negative outcomes; 4) attitudes towards physical inactivity and sedentary behaviour are diverse and depend on individual values and previous experiences; 5) perceived barriers depend on internal and external factors; 6) supporting factors are related to support and information in the social and physical environment.

**Conclusions:**

The great value that office workers attach to health is reflected in their motivations and attitudes regarding physical activity. Increasing office workers' knowledge of the health risks of prolonged sitting may therefore increase their motivation to sit less. Although ‘health’ is considered important, other values, including social and work-related values, are sometimes prioritized. We conclude that interventions that aim to reduce sedentary behaviour and increase physical activity among office workers could be improved by informing about health effects of sedentary behaviour and short/mid-term benefits of physical activity, including mental health benefits. Moreover, interventions could frame physical activity as congruent with values and support value-congruent choices. Finally, the work environment could support physical activity and interruption of sedentary behaviour.

**Supplementary Information:**

The online version contains supplementary material available at 10.1186/s12889-022-13024-z.

## Introduction

Office workers spend a significant part of their workday sitting behind their desk [1]. High levels of sedentary behaviour combined with low levels of physical activity put many office workers at increased risk for non-communicable diseases and premature mortality [2–4]. As behaviour, including physical inactivity, is driven by a complex interplay of individual and environmental factors, interventions often target individual or environmental factors to increase physical activity levels and reduce sedentary time [5–9]. Multi-component interventions targeting a range of factors appear to be most successful in changing these behaviours [6, 10, 11]. However, a dimension that is generally underemphasized in existing interventions is the role of individual perspectives, including values, that influence decision-making processes. Individuals regularly make decisions about physical activity and sedentary behaviour, for instance concerning the kind of activity, frequency of exercise, and way of commuting to work. Although some of the existing interventions targeting physical activity or sedentary behaviour have taken into account individual needs and preferences, for instance by using a participatory approach (e.g., [12, 13]), the effectiveness of interventions might be increased if even more attention is paid to individual perspectives, especially beliefs and values, in the design of interventions [14–16].

Individual values are critical motivators of behaviours and attitudes, although their impact is rarely conscious [16]. It is the relative importance of multiple values that guides individual behaviour [16]. Individual beliefs drive behaviours as well. If individuals do not believe that particular behaviour (e.g., sedentary behaviour) negatively impacts health, or if they lack this knowledge, behaviour change is unlikely. Therefore, it is essential that intervention content, and particularly educational information, confirms existing correct beliefs, corrects incorrect beliefs, and fills important knowledge gaps [17, 18].

The alignment of interventions to individuals' perspectives can support individuals in making more deliberate and autonomous choices in which they make trade-offs between different choice aspects and consider their value priorities [19–21]. Such active and value-congruent choices are considered increasingly important in public health and medical healthcare [22, 23] and are assumed to increase individuals' commitment toward health-related goals (e.g., being more physically active or less sedentary); consequently, active choices could induce more permanent behaviour change [21, 24, 25].

Studying individual perspectives related to physical activity and sedentary behaviour may provide insights into the way individual beliefs, values, motivations, and attitudes influence behavioural choices. If these insights are subsequently considered in the design of interventions, inactive individuals could ultimately be empowered to make active and value-congruent choices about their behaviour. To date, studies into individual perspectives on these behaviours have mainly focused on perceived barriers and facilitators (e.g., [26–29]). One qualitative study by Segar et al. (2017) did investigate values, beliefs, and goals regarding physical activity among women [30]. The authors reported that most women prioritized family and work over physical activity and that being physically active with others was a key ingredient for positive experiences. Moreover, many low active participants appeared to experience internal pressures to become more physically active, which often reflected a desire to obtain appearance, weight, or health benefits. To our knowledge, no previous qualitative studies have specifically investigated office workers’ perspectives on physical activity and sedentary behaviour, which are interrelated behaviours. Therefore, the current study aimed to gain insight into office workers' perspectives, including their beliefs and values, on physical activity and sedentary behaviour.

## Methods

### Study design

In this qualitative study, we conducted sixteen semi-structured face-to-face interviews with Dutch office workers. In accordance with local regulatory guidelines and standards for human subjects' protection in the Netherlands (Medical Research Involving Human Subjects Act), our study was exempted from review by Amsterdam UMC's medical research ethics committee (2019.086).

### Study participants and recruitment

We recruited office workers (e.g., in secretary, finance, or research), aged between 25 and 65, who had a mastery of the Dutch language and practiced their profession ≥ 20 h a week. Exclusion criteria, which individuals assessed themselves, included being pregnant, having an increased risk for cardiovascular diseases, suffering from a chronic disease, having a medical condition that limits physical activity, or knowing the interviewer (LL) personally. Individuals at increased risk of cardiovascular disease or suffering from a chronic disease were excluded to obtain a homogeneous sample of relatively healthy individuals. Participants were recruited through social media (LinkedIn, Facebook), printed flyers in supermarkets, universities and bank buildings in Amsterdam, the Netherlands, and two authors' networks (JJ and OD). The recruitment message specified the study aim and exclusion criteria. We initially used convenience sampling, which resulted in mostly women registering for participation. To obtain a more diverse sample with regard to gender, we adapted the recruitment flyers to recruit an additional number of men. All participants provided written informed consent.

### Procedure

One week before the scheduled interview, participants were sent a sensitizing booklet that triggered them to reflect on their physical activity and sedentary behaviour and on their values in life, which was intended to facilitate the interview [31, 32] (Additional file 1). The assignments in the sensitizing booklet asked participants to: (a) describe timelines for a regular workday and a weekend day regarding the type and level of physical activity, as well as standing behaviour and sedentary behaviour; (b) select personal values and characteristics from a list (Additional file 2); (c) select the five values or characteristics most important to them; and (d) indicate whether those five values or characteristics influence their levels of physical activity and sedentary behaviour. As from now, we will refer to values and characteristics as 'values', since the listed characteristics (e.g., 'ambition' and 'responsibility') can be regarded as values. We asked participants to bring the completed booklet to the interview as a reference. We did not ask them to hand in their booklet.

The interview guide (Additional file 3) contained predefined questions, but also allowed participants to elaborate on a particular subject. The interview started with questions about the participant's profession and working hours. Subsequently, participants were asked about their physical activity and sedentary behaviour on a regular weekday (e.g., way of commuting, stair and elevator use, sitting time at work, number and type of breaks) and on a regular weekend day. We invited participants to use their notes from the booklets to describe their physical activity and sedentary behaviour patterns. Next, questions were posed about the participant's beliefs (i.e., cognitions of the way things are related [18]) and barriers, and about supporting factors (i.e., facilitators and needs) regarding physical activity and sedentary behaviour in their social and physical environments. Probes for clarification were used to gain a deeper understanding of participants' perspectives (e.g., “Could you explain why…?”). Values (i.e., what individuals consider important in life [16]) were discussed at the end of the interview, based on the value-exercise in the sensitizing booklet. After the interview, demographics were obtained through a questionnaire.

The interview guide and sensitizing booklet were pretested by one researcher (LL) on two other researchers (JJ & OD); this resulted in minor adaptations. All interviews were conducted in March 2019 and took place at the participants' workplace or their homes. Interviews lasted 53 min on average (range: 43–66 min) and participants received a €20 gift card for their participation. After each interview, the interviewer wrote a short summary, including field notes. All interviews were conducted by the same researcher (LL), a female office worker (PhD candidate) with a background in Social Psychology and previous interviewing experience.

### Data analysis

All interviews were audiotaped and transcribed verbatim using the software package F4transkript (Audiotranskription, Germany). The short summaries and field notes were shortly analysed after each interview to see whether additional topics emerged that had not been mentioned in previous interviews. A total of sixteen interviews were conducted; no additional key insights emerged in the last four interviews, indicating that data saturation had been reached. We analysed the data in ATLAS.ti, using codebook thematic analysis, a method to identify, analyse, and report patterns in the data [33, 34]. This method allowed both inductive and deductive analysis [35].

As described by Castleberry and Nolen (2018), thematic analysis starts with *compiling*; this step consisted of data transcription and familiarization with the data. In the second, *disassembling* step, an inductive coding approach was used: four researchers (LL, ID, JJ, OD) open-coded half of the interviews (*n* = 8). The researchers stayed close to the data during this step. These codes were printed on Post-its and categorized into constructs (i.e., beliefs, values, etc.) in a meeting with the same four researchers until mutual agreement about categorization was reached. In the third step (*reassembling*), two researchers (LL, ID) used these constructs and codes to construct a structured codebook. All interviews, including the eight that were initially open-coded, were subsequently coded deductively by one researcher (LL) using the codebook. If none of the codes applied to quotes in a transcript, either the definition of an existing code was changed, or a new code was created. Such codebook adaptations were discussed within the research team to reach agreement. Themes (i.e., patterns of shared meaning, organized around a central organizing concept [34]) were organized around the identified constructs. The patterns of meaning were identified by analysing codes and corresponding data extracts. The fourth, *interpreting* step, entailed re-reading the quotations belonging to each code and describing relationships within and across themes. In the last, *concluding* step, we described our conclusions regarding participants' perspectives on physical activity and sedentary behaviour. We reported our findings in a descriptive way. To check for completeness and accuracy, we compared our conclusions with the summaries made directly after the interviews; no major deviations were observed.

## Results

### Participant characteristics

Sixteen Dutch office workers (50% female) from different professions participated. Participant characteristics are shown in Table 1. Participants' mean age was 40.7 years (SD ± 9.9); 63% were married or cohabitated; 56% had children; 88% had completed university education or professional education and all participants were born in the Netherlands. Nine participants (56%) exercised at least once a week. Participants had limited knowledge of the national physical activity recommendation, and did not know the amount of physical activity that was recommended.

**Table 1:** Participants' characteristics

**Participant ID**	**Gender**	**Age**^a^	**Marital status**	**Number of children**	**Educational attainment**^b^	**Field of profession**	**Working hours**^c^
P1	W	42	Married	2	Professional	Education	28
P2	W	25	Single	0	University	Pharmacy	40
P3	W	34	Cohabiting	1	Professional	Secretary	24
P4	W	34	Cohabiting	2	University	Research & consultancy	32
P5	W	32	Single	0	University	Research	36
P6	W	38	Single	0	Professional	Secretary	32
P7	W	32	Divorced	2	Professional	Management	32
P8	M	37	Married	2	University	Policy advisor	20
P9	M	45	Married	2	University	Finance	40
P10	M	55	Cohabiting	1	University	Finance	45
P11	M	60	Married	2	Professional	ICT	40
P12	M	38	Cohabiting	0	Professional	Finance	40
P13	M	41	Cohabiting	0	Professional	Finance	44
P14	M	62	Married	0	University	Finance	40
P15	W	54	Divorced	3	Secondary	Secretary	25
P16	M	42	Relationship	0	Secondary	ICT	36

Abbreviations: *W* woman, *M* man, *ICT* Information and communications technology

^**a**^ Age in years

^b^ *Secondary*: Secondary education including higher school or leaving certificate/trade/apprenticeship; *Professional*: professional education including certificate/diploma; *University*: university education including university degree or higher

^c^ Average number of working hours per week

### Thematic analysis

We identified six main themes in our qualitative data, which related to participants' perspectives on physical activity and sedentary behaviour. The themes are described in the next section, illustrated by quotes.

#### Theme 1: Beliefs about health effects are specific regarding physical activity, but superficial regarding sedentary behaviour

When asked about the consequences of insufficient physical activity, all participants mentioned health risks (i.e., *hypertension*, *high cholesterol levels*, *arteriosclerosis*, *diabetes*, *cardiovascular diseases, cancer*).

[About the health risks of physical inactivity] "I think it increases the risk of cardiovascular diseases; and cholesterol levels; I think that your endurance will decrease when you're continuously sitting and if your endurance decreases, you might experience some form of mental imbalance earlier. I also think that the risk of other diseases increases because it weakens your immune system. [P14]

Furthermore, they all explained that physical activity benefits *physical* health (i.e., *increased endurance*, *prevention of weight gain*, *reduction of stiffness*, *increased blood circulation*, and *positive effects on muscles and joints*) and most participants also mentioned *mental* health benefits (i.e., *higher energy levels*, *better resistance to stress*, *increased relaxation* and *a general feeling of satisfaction*).

I: "What do you like about going home by bike in the afternoon?"

P: "Well, the fresh air, I love being outside and it also gives me the chance to process my daily impressions, which makes me more relaxed when I arrive home." [P6]

Regarding sedentary behaviour, participants' beliefs were more superficial; almost all participants mentioned that too much sitting is 'bad' for one’s health, often referring to the popularized media statement 'Sitting is the new smoking'.

“Sometimes you hear ‘sitting is the new smoking’, but I haven't really figured out why that is the case exactly, so to be honest I don't know whether it has to do with your sitting position or because you’re not physically active, but I've heard this saying so often that I assume it’s true.” [P8]

Still, most participants did not know *which* short- and long-term health effects were associated with high levels of sitting. A few participants were able to specify possible physical health effects (i.e., *increased stiffness or pain in the neck, shoulders or back, weight gain*, *reduction of strength and endurance,* and *reduced blood circulation*) or mental health effects (i.e., *tiredness* and *a reduced focus*), mostly relating to short-term effects.

#### Theme 2: In addition to ‘health’ as a value, other values are also given priority

When asked about general values in life, participants frequently mentioned *health*, *family*, *friendships, balance,* and *freedom.*

"A 'good life'… is being conscious right now as well as about the future, both for physical activity and healthy diet, as well as with your living circumstances." [P16]

"The urge to grow old and stay healthy, yes, I think that’s what I am doing indirectly, well, to be happy you need to be healthy." [P9]

Some of those values, especially health, family, and friendships, were also discussed to some extent when talking about motivations and attitudes. The value *health* was frequently mentioned as a driver of physical activity, but other values were also mentioned in relation to physical activity, either directly (e.g., exercise for *pleasure*), or indirectly (e.g., being active in order to age healthily to stay with one's *family*). Sometimes, participants were ambivalent about values relating to physical activity. In most of these cases, engaging in physical activity for health reasons conflicted with social values (i.e., *family*, *friendships*) or with work-related values (i.e*.*, *ambition*, *responsibility*, *performance*, *money*). For instance, several participants wanted to be physically active after work but chose to spend evenings at home, because spending time with family was also considered important.

I: "Do you see opportunities at work to be more physically active or to sit less?"

P: "We have exercise facilities [at work] but I don’t feel like going for a quick workout during lunch or before or after work; after work I want to go home, eat and see my children." [P9]

#### Theme 3: Motivations to engage in physical activity mainly stem from prioritizing the value ‘health’, reflected by a desire to both achieve positive short/mid-term outcomes and to prevent long-term negative outcomes.

'Good health' was a frequently mentioned motivation and reflected the value 'health'. When asked to clarify, participants specified their health-related motivations, which typically referred to short and mid-term positive outcomes (e.g., *increasing endurance*, *keeping fit, having a healthy body weight*, *feeling good*), and occasionally to longer-term outcomes (e.g., *maintaining a healthy body weight* and *being physically active at an older age*). Other positive outcomes that participants claimed to strive for included *having fun, attaining specific achievements* (e.g., walk 10,000 steps a day) and *looking good*.

"Actually, I think I should exercise at least three to four times a week, just because it’s better, it’s healthier, and it may result in a good body shape [laughs]. You just want to keep looking good while aging, and maintain good endurance and good health." [P6]

[About the motivation to exercise] "I think that my biggest motivation is to look fit and to be fit." [P2]

Most motivations to engage in physical activity stemmed from a desire to achieve the aforementioned *positive* outcomes, indicating positive valence. Occasionally, motivations stemmed from a desire to prevent *negative* outcomes, indicating negative valence. Motivations to prevent negative outcomes were typically related to long-term consequences (e.g., *gaining weight*, *getting physical complaints,* and *becoming ill)*.

[About the motivation to exercise] "Currently, I am too heavy for my height, so yes, that was certainly an important motivation to work on it" [P7]

Motivations discussed by participants concerning sedentary behaviour generally applied to the short term (e.g., *reduce stiffness*, *move away from the computer screen*, *regain a better focus*). This short-term focus was also reflected in participants' beliefs about sedentary behaviour (theme 1).

#### Theme 4: Attitudes towards physical inactivity and sedentary behaviour are diverse and depend on individual values and previous experiences

Participants expressed diverse attitudes (i.e., favourable or unfavourable evaluations of the behaviour in question [36]) toward physical activity, including exercise. Many positively evaluated physical activities due to positive previous experiences, or because certain activities corresponded with their underlying values; for instance, participants who valued *social connections* highly enjoyed playing team sports.

[About table tennis] "I really like this sport, I really enjoy it, […] the game is really fun and the people are very nice as well." [P3]

Sometimes, participants expressed a neutral or somewhat negative attitude toward a certain physical activity (e.g., fitness or running); some of them still engaged in the activity for health reasons, reflecting *health* as a value.

I: "Do you enjoy going to the gym?"

P: "Well let's say it’s not my favourite hobby, it’s not the most challenging thing I can think of… maybe I would rather have a home trainer in my apartment (laughs), although it’s important for me; I know it’s beneficial for my health; I’m not the kind of person who stays at home when I have an off day; I will go just because it’s important [to exercise]." [P12]

I: "You mentioned previously that you don’t like doing sports; have you never enjoyed sports?

P: "Well, I’m not very good at game-type sports […], but I did enjoy lessons like aerobics; it helped me to build up my muscles and it made me enthusiastic. Currently, however, it’s difficult to combine with work and my children; for sports lessons I have to be somewhere at a fixed time, and then I feel obliged and stressed to be there; so I can’t really enjoy it." [P4]

Participants also expressed diverse attitudes toward sedentary behaviour. Negative previous experiences with prolonged sitting, such as physical complaints in neck and shoulders, were mentioned in relation to a negative attitude toward prolonged sitting. For some participants, this seemed to result in a positive attitude toward alternating between sitting and standing postures at work. Other participants had a more negative attitude toward alternating since they experienced tiredness when working in a standing posture. Finally, attitudes seemed to depend on the situation; attitudes toward sitting were more positive when participants had already done something that required physical activity (e.g., exercising or cleaning the house), or when sitting was accompanied by a relaxing activity (e.g., watching television).

#### Theme 5: Perceived barriers depend on internal and external factors

All participants indicated one or multiple barriers regarding physical activity or reduction of sedentary behaviour. Perceived barriers were either related to internal factors (i.e., *lack of motivation/energy/inspiration* or *physical complaints*) or external factors in the physical or social environment (i.e., *bad weather*, *lack of childcare* or *lack of social support*). Lack of time was a frequently mentioned barrier to physical activity and was mainly caused by competing interests (described in theme 2).

"I used to engage in sports quite often, but now I lack time and energy. It’s also due to the informal care I’ve been providing for a long time; I couldn’t combine the two any longer." [P16]

Some participants mentioned more barriers for physical activity than others; this seemed to be related to participants' values (i.e., more barriers were perceived when competing interests played a role) and the degree of motivation for physical activity (i.e., more barriers were perceived in the case of little motivation).

Perceived barriers regarding interruption of sedentary behaviour at the workplace often concerned high work pressure, inflexible work policies, lack of facilities such as sit-stand desks and unfavourable social norms (e.g., no other colleagues working in a standing position).

"Sometimes colleagues go for a short walk and invite others to join; I mostly say no due to work pressure" [P5]

[About sit-stand desks] "In general I think that those things are contagious when you see others using them; then you think 'oh let's also try that once', while if nobody's ever using it, you don’t want to be the only idiot using it." [P8]

#### Theme 6: Supporting factors are related to support and information in the social and physical environment

Participants mentioned factors that *supported* them in being physically active and less sedentary, and factors that *would support* them in these behaviours. Supporting factors mentioned regarding the social environment included positive social norms (e.g., relating to working in a standing position), social activities that require physical activity, and social support from the social environment, including encouragement. Some participants wished to receive more social support.

I: "How could your social environment support you to be more physically active?"

P: "It would help me if others invited me to come along […]; it would support me if my partner and I exercised together; I would really enjoy that" [P3]

Regarding the physical environment, an inviting environment, good weather conditions, activity trackers, and owning a pet were generally considered to encourage physical activity and less sedentary behaviour. At work, multiple participants wanted to have a sit-stand desk or more physical activity, including active breaks.

"The availability of facilities [at work], such as sit-stand desks and meetings rooms with a standing table, would support me [to stand up more often]" [P8]

Finally, multiple participants stressed that employers have a responsibility in encouraging physical activity and discouraging sedentary behaviour. Some participants wished to regularly receive advice about easily applicable active behaviours, for instance through e-mails or newsletters from their employer, and some participants wanted to receive more information about the health effects of physical activity and interruption of sedentary behaviour, for instance through messages in a 'did-you-know-that…' format at the workplace.

"That’s a role for employers [to provide information about physical activity and sedentary behaviour], because an employer wants his employees to be in good health […], so it’s beneficial if employers play a role in this. Either by increasing awareness or by facilitating equipment for their employees to be more physically active" [P13]

## Discussion

Our study aimed to gain insight into office workers' perspectives on physical activity and sedentary behaviour and, in particular, their beliefs and values. Our thematic analysis revealed six main themes. We found that participants’ beliefs about health effects were specific regarding physical activity, but superficial regarding sedentary behaviour. We also found that in addition to ‘health’ as a value, other values, including social and work-related values, were also given priority. The remaining themes related to participant’s motivations and attitudes, and to perceived barriers and supporting factors.

In line with psychological literature [16, 37–40], the beliefs, values, motivations, attitudes, barriers, and supporting factors mentioned by office workers in our study were interrelated. For instance, we found that individual *values,* such as ‘health’, ‘family’, and ‘pleasure’*,* were related to *motivations* to be physically active, and that *attitudes* toward certain activities were positive if those activities corresponded with underlying *values*. Furthermore, our results showed that participants perceived more *barriers* to physical activity if their *motivation* was low or if they had conflicting *values*. The latter is also reflected in previous studies among women, not specifically office workers, for whom family and caregiving responsibilities presented a major barrier to physical activity [30, 41]. Together, these findings suggest that there is room for improvement of interventions by fostering awareness of potential inconsistencies between values in office workers (e.g., conflicts between work and health-related values), and by linking physical activity goals to values such as health, family, and pleasure.

An interesting finding was that participants’ beliefs about sedentary behaviour were superficial, and that their knowledge about consequences of sedentary behaviour was limited, especially in the long term. This finding is consistent with previous studies that examined beliefs about sedentary behaviour [42, 43]. A possible explanation is the relatively recent scientific and public interest in excessive sedentary behaviour [44]. Moreover, public health guidelines lack specific recommendations regarding sedentary behaviour until now, due to a lack of evidence, and because sedentary behaviour and physical activity interact [45–48]. More precise recommendations about sedentary behaviour that are disseminated in an appropriate way could increase individuals’ knowledge. Moreover, educating office workers about the potential health consequences of prolonged sedentary behaviour may motivate them to reduce sitting time.

Another main finding was that participants occasionally experienced ambivalence between the value 'health', which was strongly related to physical activity, and social or work-related values. Participants also indicated that, as a consequence, this sometimes led them to engage less in physical activity than they intended. Segar et al. (2017) also reported that family and work were often prioritized over physical activity, although study participants were not specifically office workers [30]. As it is thought that the relative importance of multiple values guides behaviour [16], it is tempting to assume that individuals' behaviour (e.g.: working long days and barely engaging in physical activity) reflects the relative importance of their values. However, this may not be the case when individuals do not actively consider the relative importance of their values. Supporting individuals in clarifying their values and resolving ambivalence can help individuals make active choices about their behaviour that are in accordance with their most important values [49]. This is assumed to increase individuals’ commitment toward the chosen behaviour (e.g., becoming more physically active/ less sedentary). Consequently, such choices may induce more permanent behaviour change [21, 24, 25].

The finding that participants' motivations to engage in physical activity often concerned short and mid-term outcomes is also consistent with previous studies [50–52]. Previous studies reported short-term *mental* and *affective* outcomes as key motivators; however, in contrast to our findings, short-term *physical* health outcomes seemed to be less important in motivating physical activity [50–52]. Participants' predominant focus on short-term outcomes may be caused by a general tendency to give more relative weight to payoffs closer to the present time when considering trade-offs between two future moments. This 'present-biasedness' [53] has previously been associated with physical activity behaviour [54].

Finally, our findings showed that contextual factors played a role in participants’ choices regarding physical activity and interruption of sedentary behaviour. For instance, sit-stand desks and positive social norms at the workplace were mentioned as supporting factors for reducing sedentary behaviour, whereas high work pressure and a lack of facilities were perceived as barriers. These contextual factors have also been reported in previous studies among office workers (e.g., [29, 55, 56]). The contextual factors mentioned often appeared to play a role in participants’ behavioural choices at work independently of their values, beliefs, and motivations. Therefore, the workplace, including its physical and social environment, appears to be an important setting for health promotion.

### Strengths and limitations

The use of sensitizing booklets may have contributed to a deeper understanding of values and behaviour, as the booklets allowed participants to reflect on their values and behaviour prior to the interview [31, 32]. However, our study was also subject to a few limitations. Although qualitative research is not primarily concerned with obtaining generalizable results [57], it should be noted that two profession fields, i.e., finance and secretary, were overrepresented in our sample. Moreover, convenience sampling and use of a recruitment flyer that revealed the study focus may have resulted in a relatively high proportion of participants with interest in physical activity. However, the proportion of participants who exercised at least once a week corresponded to the proportion in the Dutch adult population aged 18–64 years [58]. Finally, our sample lacked diversity regarding ethnicity and educational level.

### Practice implications

To ensure that interventions are more closely aligned with office workers' perspectives, educational information could have a more explicit emphasis on short-term positive effects, including mental health effects [52, 59]. Furthermore, it is essential to inform office workers about the health effects of prolonged sitting, as this appeared to be an important knowledge gap [18]. Interventions could include tools such as Motivational Interviewing [60–62] or the Disconnected Values (Intervention) Model [63–66] to support office workers in clarifying their values relating to physical activity and sedentary behaviour, which would empower them to make more active and value-congruent choices.

From a health-promotion perspective, an implication would be to frame physical activity messages so that conflicting values are viewed as being congruent, rather than conflicting, with physical activity. Segar et al. (2017) proposed developing physical activity messages that emphasize that time spent on physical activity can contribute to family responsibilities and that physical activity can be a way to connect with others [30].

Based on the barriers and supporting factors identified, employers seem to have a role in discouraging sedentary behaviour and promoting physical activity among office workers during working hours. Employers could provide equipment (e.g., sit-stand desks) to shape the work environment and implement interventions that provide information about the health effects of prolonged sedentary behaviour and advice about easily applicable active behaviours or active breaks. Although this recommendation may seem evident and employers increasingly pay attention to this [67], there still seems room for improvement. These implications also apply to situations where office workers work from home, which is currently the case for many office workers because of the COVID-19 pandemic.

## Conclusions

The great value that office workers attach to health is reflected in their motivations and attitudes regarding physical activity. Increasing office workers' knowledge of the health risks of prolonged sitting may therefore increase their motivation to sit less. Although ‘health’ is considered important, other values, including social and work-related values, are sometimes prioritized. Interventions that aim to reduce sedentary behaviour and increase physical activity among office workers could be improved by providing information about health effects of sedentary behaviour and short/mid-term benefits of physical activity, including mental health benefits. Moreover, interventions could frame physical activity as congruent with values and support value-congruent choices. Finally, it is important that the work environment supports physical activity and interruption of sedentary behaviour.

## Supplementary Information

**Additional file 1.** Booklet.

**Additional file 2. **Substantiation of the list of values and characteristics.

**Additional file 3.** Topic guide.

## Data Availability

The data generated during the current study are not publicly available due to content that could compromise research participant privacy but are available from the corresponding author on reasonable request.
