# Supplementary material for: Office workers' perspectives on physical activity and sedentary behaviour: a qualitative study
Source: BMC Public Health. 2022 Mar 30;22:621. doi: 10.1186/s12889-022-13024-z (PMC8966601; doi:10.1186/s12889-022-13024-z)

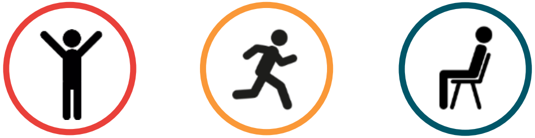

Booklet

This booklet serves as a preparation for the interview about physical activity and sedentary behaviour

Why this booklet?

Dear participant,

Thank you for you willingness to participate in an interview about physical activity and sedentary behaviour. This interview is part of a scientific study. The aim of this study is to gain more insight into people's motivations for physical activity and sedentary behaviour, and to acquire knowledge about what people consider important in this regard.

To prepare you for the interview, we ask you to complete the 3 assignments in this booklet.
This will take about 15 minutes.

Could you please bring the booklet to the interview?
We will use your answers in this booklet as a starting point in the interview.
You do not need to return the booklet afterwards.

There are no right or wrong answers. Please be honest about your behaviour.
Everything you tell us in the interview is valuable for our study.
If you have any questions, please do not hesitate to contact me:

[Blinded contact details of the interviewer]

Practical information

- *Interview date:*

  ……………………………………………………………

- *Interview duration: ± 45 minutes*
- *Reimbursement: a VVV gift voucher worth €20*

Assignment 1: Describe your workday

Please select a **workday** next week and describe what this day looks like from the morning until the evening. For each activity, describe whether you are sitting, standing or moving (and the type of movement, such as "climbing stairs" or "cycling"). Please write down the time of your activities above the timeline.

Time:

....	....	....	....	....	....
Getting up	Going to work	Lunch	End of workday	Dinner	Bedtime

How do you commute to work?

....

....

Do you sit, stand or walk during lunch?

....

What are you doing after work? (please describe sitting, standing and moving behaviour)

....

....

....

....

....

What are you doing at work? (please describe sitting, standing and moving behaviour)

....

....

....

....

....

Assignment 2: Describe your weekend day

Please select a **weekend day** next week and describe what this day looks like from the morning until the evening. For each activity, describe whether you are sitting, standing or moving (and the type of movement, such as "climbing stairs" or "cycling"). Please write down the time of your activities above the timeline.

Time:

....

Getting up | Breakfast | Lunch | Dinner | Bedtime

What are you doing this day? (please describe sitting, standing and moving behaviour)

....

....

....

....

....

....

Assignment 3: What is important to you?

A list with general characteristics and values is presented below. This assignment consists of 3 steps:

Step 1: Please circle the characteristics and values that apply most to you as a person (select up to 15).

Step 2: From the characteristics/values that you circled, please select 5 characteristics/values that are most important to you; indicate them with an arrow (→).

Step 3: For each of these 5 characteristics/values, indicate whether they (directly or indirectly) influence your levels of physical activity and sedentary behaviour. If they do, indicate this with a plus (+) ; if they do not, indicate this with a minus (–).

- Ambition
- Autonomy
- Balance
- Helpfulness
- Proficiency
- Politeness
- Creativity
- Family
- Money
- Health
- Learning
- Charity
- Nature
- Independence
- Entrepreneurial
- Personal growth
- Pleasure
- Achievement
- Religion
- Being together
- Sensation
- Spirituality
- Spontaneity
- Stability
- Status
- Challenge
- Responsibility
- Friendships
- Freedom
- Certainty
- Self-discipline

Notes

Use this space for any notes you wish to make.

Thank you!

Please remember to bring this booklet to the interview

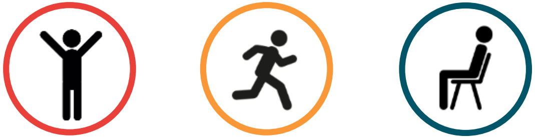

Supplement: Supplementary file 1 — Additional file 1. Booklet. [file 12889_2022_13024_MOESM1_ESM.pdf]
