# Supplementary material for: Office workers' perspectives on physical activity and sedentary behaviour: a qualitative study
Source: BMC Public Health. 2022 Mar 30;22:621. doi: 10.1186/s12889-022-13024-z (PMC8966601; doi:10.1186/s12889-022-13024-z)
Supplement: Supplementary file 2 — Additional file 2. Substantiation of the list of values and characteristics. [file 12889_2022_13024_MOESM2_ESM.docx]

**Additional file 2. Substantiation of the list of values and characteristics**

In this document, we substantiate the values and characteristics that were included in the list of the third assignment of the sensitizing booklet.

We started with a list of general values and characteristics that one of the research team members (OD) had received during a training course. Next, we added values and characteristics that were mentioned during the pilot interviews, and added some values from Schwartz’s theory of basic values [1]. We subsequently selected those values and characteristics that we (as a research team) deemed important (either directly or indirectly) in relation to physical activity and/or sedentary behaviour. We then categorized these values and characteristics into the four dimensions of values described by Schwartz (2012) [1]:

1. Self-enhancement
2. Openness to change
3. Conservation
4. Self-transcendence

This resulted in the following structure:

| 1. **Self-enhancement**  - Ambition - Proficiency - Money - Learning - Personal growth - Achievement - Status - Pleasure | 1. **Openness to change**  - Entrepreneurial - Sensation - Spontaneity - Challenge - Freedom - Independence - Autonomy - Creativity |
| --- | --- |
| 1. **Conservation**  - Family - Religion - Being together - Stability - Certainty - Health - Self-discipline - Politeness | 1. **Self-transcendence**  - Family - Charity - Nature - Responsibility - Friendships - Balance - Spirituality - Helpfulness |

These values and characteristics were included in the third assignment of the sensitizing booklet.

***References***

1. Schwartz, S.H., *An overview of the Schwartz theory of basic values.* Online readings in Psychology and Culture, 2012. **2**(1): p. 11.
