# Supplementary material for: Office workers' perspectives on physical activity and sedentary behaviour: a qualitative study
Source: BMC Public Health. 2022 Mar 30;22:621. doi: 10.1186/s12889-022-13024-z (PMC8966601; doi:10.1186/s12889-022-13024-z)
Supplement: Supplementary file 3 — Additional file 3. Topic guide. [file 12889_2022_13024_MOESM3_ESM.docx]

**Additional file 3. Topic guide**

**Introduction**

- **Introduction by researcher**
- **Participant signs informed consent**
- **Researcher explains the structure of the interview**
- **Diary**
  - Did you receive the diary in preparation of this interview?
  - Were you able to complete this diary?
- **Profession:** Before we continue with the description of your workday, I would like to know a little more about your current profession
  - **Current profession**
  - **Number of working days**
  - **Number of working hours**

**Physical activity and sedentary behaviour during a workday**

1. I would like to continue by talking about the workday you described in your diary.
   - How did you commute to work on that particular day?
   - On which floor of the building do you work? (prompt: Did you take the stairs or the elevator?)
   - What did you do during your working hours? How much did you actually move, stand or sit throughout this workday?
   - During lunch did you sit, stand, or did you go out for a lunchtime walk?
   - What did you do during leisure time after a workday? How much did you actually move, stand or sit throughout leisure time?

**Participants’ choices regarding physical activity and sedentary behaviour during the workday. Examples of in-depth questions:**

1. Why did you choose ….? (goals)

Did you consider an alternative? Why didn’t you choose this alternative? Why did you finally choose for…? To what extent did you make this choice consciously?

**In the case of a habit:**

1. How has this become a habit?

**Determining the underlying motivations and values of physical activity or sedentary behaviour**

1. Why/for what reason…?
2. Why is this important for you?
3. How does this description differ from an average working day in terms of physical activity and sedentary behaviour?

**Physical activity and sedentary behaviour during a weekend day**

**The constructs are structured in the same way as for the workday.**

1. We talked about your workday, but I am also interested in the weekend day you described in your diary.
   - Could you describe what you did in the morning?
   - Could you describe what you did in the afternoon?
   - Could you describe what you did in the evening?
2. How does this description of your weekend day differ from an average weekend day in terms of physical activity and sedentary behaviour?

**Beliefs**

**Determining underlying beliefs in terms of physical activity and sedentary behaviour**

1. What are the benefits of… [behaviour]?/ What are the advantages of… [behaviour] for you/for your health?
2. What (possible) disadvantages does… [behaviour] have for you/for your health?
3. What possible health consequences could there be for someone who does little or no physical activity? (**prompt**: Any negative consequences? Or do you consider it unimportant? Could you explain why you think this is the case and what effect this has on your body?)
   - How much physical activity does someone of your age need per week (according to experts)? Do you think you comply with these guidelines?
   - Is it important to you to comply with the guidelines? Why?
4. Could you tell me what the possible health consequences are for someone who remains seated for long periods of time? (**prompt:** Any negative consequences? Or do you consider it unimportant? Could you explain why you think this is the case and what effect this has on your body?)
5. Do you think your sedentary behaviour could have negative consequences for your health?

**Determining underlying beliefs in terms of the specified alternative option(s)**

1. What are the advantages of… [behaviour] for you/your health?
2. What are the (possible) disadvantages of… [behaviour] for you/your health?

**Goals, preferences, barriers, facilitators, needs, values
Physical activity**

1. How satisfied are you in general about the extent to which you engage in physical activity? Would you like to change something about your behaviour?
   - (You can be honest with me, you do not have to say you want to be more physically active because you are having this interview. We are also interested in reasons why people do not want to change their behaviour).
2. Can you tell me why you want/don’t want to change this? (Sometimes there are multiple reasons or the reason is not obvious; you can take your time to think about this question). **(health values)**
3. Why is this important/less important to you? Why do you value this? **(health values)**

If the participant wants to change something:

1. How much (more) do you want to engage in physical activity? **(goal)**
2. What type of physical activity/exercise would you like to do? **(preference)**
3. Which factors hinder you from being physically active? **(barriers)**
4. Which factors facilitate you to be physically active? **(facilitators)**
5. What do you need to achieve your goals regarding physical activity? **(needs)**
   - How could your social environment help you to be more physically active?
   - How could your physical environment help you to be more physically active?
   - Would you like to receive more information about the health effects of physical activity or inactivity? If so: what kind of information would you like to receive and how?

**Sedentary behaviour**

1. How satisfied are you in general about the extent to which you engage in sedentary behaviours? Would you like to change something about your behaviour?
   - (You can be honest with me, you do not have to say you want to be less sedentary because you are having this interview. We are also interested in reasons why people do not want to change their behaviour).
2. Can you tell me why you want/don’t want to change this? (Sometimes there are multiple reasons or the reason is not immediately obvious; you can take your time to think about this question). **(health values)**
3. Why is this important/less important to you? Why do you value this? **(health values)**

If the participant wants to change something:

1. How much time would you like to sit throughout the day? **(goal)**
2. What factors currently hinder you from reducing your sedentary behaviour? **(barriers)**
3. What factors facilitate you to interrupt your sedentary behaviour? **(facilitators)**
4. What do you need to achieve your goals regarding sedentary behaviour? **(needs)**
   - How could your social environment help you to be less sedentary?
   - How could your physical environment help you to be less sedentary?
   - Would you like to receive more information about the health effects of sedentary behaviour? If so: what kind of information would you like to receive and how?

**Values and characteristics**We talked about your physical activity and sedentary behaviour, including what you think is important. I am also curious about other key values that are important to you. In assignment 3 of your diary we provided a list of values and characteristics and asked you to identify the values and characteristics that are most important for you. Did you manage to complete this assignment?

1. Which values and characteristics did you select in the first step of the assignment?
2. Which five values/characteristics are most important to you?
3. Which values/characteristics – directly or indirectly – influence the extent to which you are physically active or sedentary? Can you explain why?
4. What characterizes a ‘good life’ according to you?

**End of the interview**

1. Would you like to add anything we have not yet discussed but might be of relevance for this interview?
2. Do you have any further questions?

Thank you very much for your participation in this interview. Your answers are valuable and may be used to improve lifestyle programs that are in line with people’s values and needs.

- - **Participant completes the questionnaire about demographic information**
  - **Participant completes the form that requests permission of contact details for further studies**
  - **Researcher hands over the reimbursement for participation (VVV gift voucher) to the participant**
